# Observed and projected changes in the climate based decay hazard of timber in the United Kingdom

**DOI:** 10.1038/s41598-020-73239-1

**Published:** 2020-10-01

**Authors:** S. F. Curling, G. A. Ormondroyd

**Affiliations:** BioComposites Centre, Bangor University, Bangor, LL57 2UW Gwynedd UK

**Keywords:** Microbiology, Materials science, Climate-change impacts

## Abstract

Current climate projections suggest that the UK will experience warmer and wetter winters and warmer and dryer summers. This change in the climate could affect the incidence or severity of microbiological attack on exposed timber and have significant impact on buildings and construction. One method of assessing the geographical climate based hazard is to use the Scheffer Climate index, which relates temperature and rain variables. There was a considerable increase in the Scheffer climate indices for various locations of the UK from 1990 to 2019. The highest index values are seen in the Northern and western areas of the United Kingdom, but increases are seen across the country. The paper also uses representative concentration pathway (RCP) scenarios to project future climate decay indices for the United Kingdom until the end of the twenty-first century. The projections show an increase in the Scheffer index even in the lowest RCP scenario, with indices in all regions of the UK increasing to indicate very high hazard of decay. The major implication is that to ensure serviceability of wood and wooden structures exposed to the environment the use of good design, durable woods and properly treated or modified woods will be paramount.

## Introduction

If the conditions are favourable, biological materials face an inevitable risk of decay and deterioration from the action of other organisms. Timber can be attacked by a variety of organisms such as fungi, bacteria and insects, with decay causing fungi a major source of economic loss. Decay fungi require certain conditions—an adequate temperature and a moisture source—to enable them to decay wood. Inside structures, these aspects can be controlled but for exterior timber out of ground contact, these factors are determined by the climatic conditions the wood is exposed to. A number of models have been used to determine climate risk and service life^1–6^ but one of the earliest and simplest is the climate index developed by Theodore Scheffer in 1971^7^ and termed the Scheffer Climate Index. The Scheffer Climate Index (SCI) determines regional risk for decay based on mean temperature and the number of days where rain is greater than a prescribed value. This index was originally determined for the contiguous states of the USA but has since been used in other regions e.g. Canada^8^, Europe^6,9^ and Korea^10^. The modelling performed has shown that the UK (particularly western areas) and Ireland lie in zones that have some of the highest SCIs in Europe^6,11^. Whilst there has been some good correlation between the SCI and decay occurrence^8^ it is not a perfect measure of decay likelihood due to local climate variables^12^. However, in terms of general regional trends and hazard mapping the SCI is a useful tool. As decay requires optimum temperature and moisture it is likely that it will be affected by climate change, with predictions stating that temperature and precipitation will rise in the UK in the future. The SCI is a good tool for tracking this change and previous work has shown that the index is rising in the US and Canada^8,13,14^ and in Korea^10^. In the UK, modelling of the central region data in 2006^15^ shows an expected rise in the SCI in the future. Projections of the climate decay index have been performed for Oslo and Bergen in Norway and indicate an expected increase in the two cities modelled with indices during the winter months in particular, increasing substantially^16^. Previous modelling of the UK SCI^17^ utilised United Kingdom Climate Projection 2009 (UKCP09) data^18^. This paper utilises more up to date United Kingdom Climate Projection 2018 (UKCP18) data^18^ which covers a wider range of climate scenarios. These UKCP 18 projections are in turn based on four Representative Concentration Pathway (RCP) scenarios. The main premises of these scenarios are briefly described below;

- RCP 2.6: This is a peak and decline model with peak values encountered mid-century and then dropping by 2100. This model relies on substantial climate change mitigation efforts^19^.
- RCP 4.5: A stabilisation scenario with effects peaking at 2100 with some mitigation on emissions^20–22^.
- RCP 6.0: A high emissions scenario peaking at 2100^23,24^
- RCP 8.5: A high emissions scenario often used as the “worst case” or “business as usual” scenario^25^. However, existing mitigation efforts may have made RCP 8.5 increasingly unlikely and therefore less useful^26^.

This paper uses historic data from the UK meteorological office to determine the historic and current Scheffer index for different regions of the UK; how they have changed between 1960 to 1990 and 1990 to present (2019); and to estimate future SCI following climate change scenarios.

## Results and discussion

### Historic and current data

The tridecadal data (based on 30/29 years) for all regions as determined in 1990 and 2019 is shown in Table 1. In all areas there was an increase in the index (shown as the percentage change) from 1990 to 2019. In the majority of cases the data shows statistical significant difference at the traditional p = 0.05 confidence level as determined by t-test (actual p values are shown in Table 1).

**Table 1:** SCI index for 1990 and 2019 based on mean 30 year data.

Area	1990 30 Year SCI	2019 30 Year SCI	Change (%)	p-value
North Scotland	82.60	99.46	20.41	0.003
East Scotland	73.88	86.29	16.80	0.002
West Scotland	89.59	110.0	22.78	0.03
Northern Ireland	84.17	94.85	12.69	0.08
North West England and North Wales (NW)	82.15	95.73	16.52	0.019
East and North East England (NE)	72.27	86.75	20.03	0.0005
Midlands	72.22	85.66	16.60	0.01
East Anglia	74.34	86.81	16.78	0.003
South East and South Central England (SE)	71.15	84.10	18.21	0.0005
South Wales and South West England (SW)	89.00	104.90	16.81	0.0007

The decadal and tri-decadal data are shown graphically in Figs. 5 and 6 (denoted as historical data) for the regions exhibiting the highest (West Scotland) and lowest (South East England) climate indices. The data shows a general steadily fluctuating value of SCI until approximately the year 2000 when there appears to be the start of a steady and significant increasing SCI trend. In line with previous work^8,14^ the data shows a significant change in the SCI and the associated decay hazard over the past 30 years. This implies that the decay hazard for exterior above ground wood exposure has also increased, although when using the SCI it must be remembered that localised conditions ultimately determine the decay hazard.

The data in Table 1 shows that all regions have an index rating of high hazard (SCI 65–100) in 1990. By 2019 in two of the 10 regions, West Scotland and South West England, the hazard had increased to the very high hazard category (SCI 100+).

### Scheffer Climate Index projections

**Figure 1:**
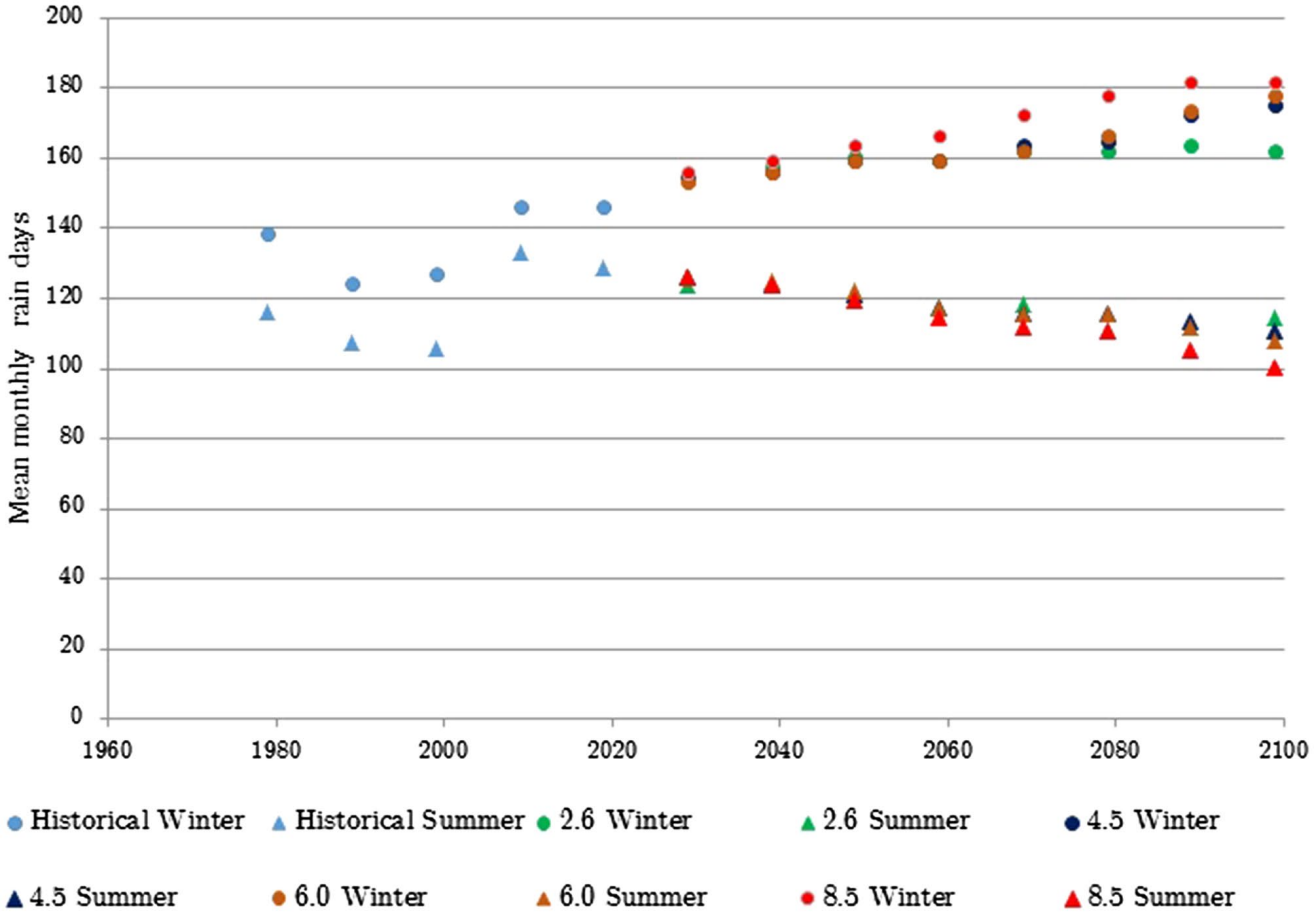
Observed and projected winter and summer (under RCP 2.6. 4.5, 6.0 and 8.5 scenarios) seasonal number of rain days for the West Scotland region.

**Figure 2:**
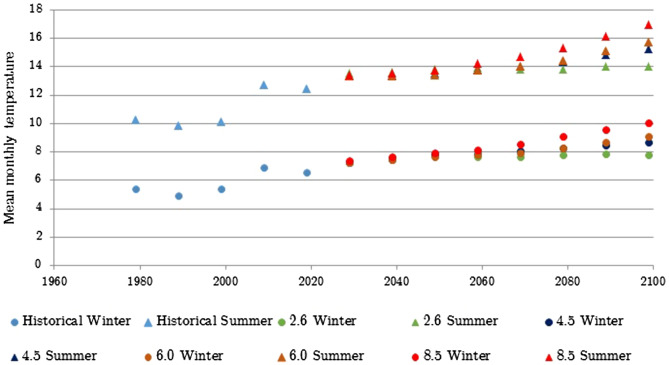
Observed and projected winter and summer (under RCP 2.6. 4.5, 6.0 and 8.5 scenarios) mean monthly temperatures for the West Scotland region.

**Figure 3:**
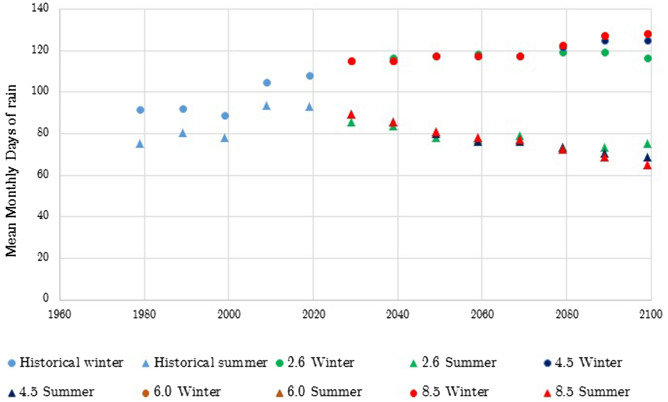
Observed and projected winter and summer (under RCP 2.6. 4.5, 6.0 and 8.5 scenarios) seasonal number of rain days for the South East England region.

**Figure 4:**
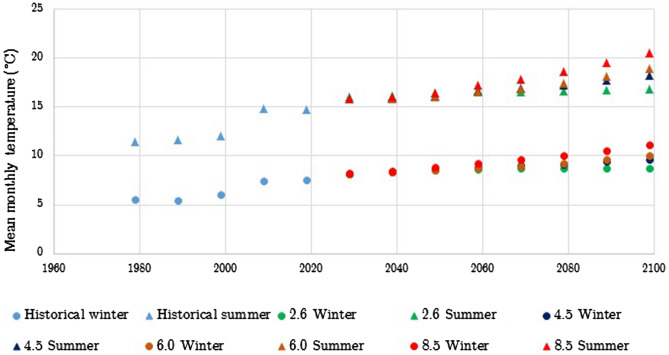
Observed and projected winter and summer (under RCP 2.6. 4.5, 6.0 and 8.5 scenarios) mean monthly temperatures for the South East England region.

Climate projections made based on the 2019 data and the UK met office projections were used to project future temperature and days of rain for all regions. For brevity the graphical presentations of the mean monthly temperature and mean seasonal number of rain days for the West of Scotland and South East England (as the regions with the highest and lowest Scheffer indices) are shown in Figs. 1, 2, 3, 4. In the projections the number of rain days for RCP 8.5 is capped at 182 as this is the maximum number of days in the season.

The historical part of the data shows that the mean monthly temperature has been steadily rising for approximately the past 20 years, during both winter and summer. The data also shows an increase in winter rain that is projected to increase. For the summer season the historical data has shown an initial increase in summer rain days between 2000 and 2010 and then a decrease until 2019. This decreasing trend is projected to continue under all scenarios.

**Figure 5:**
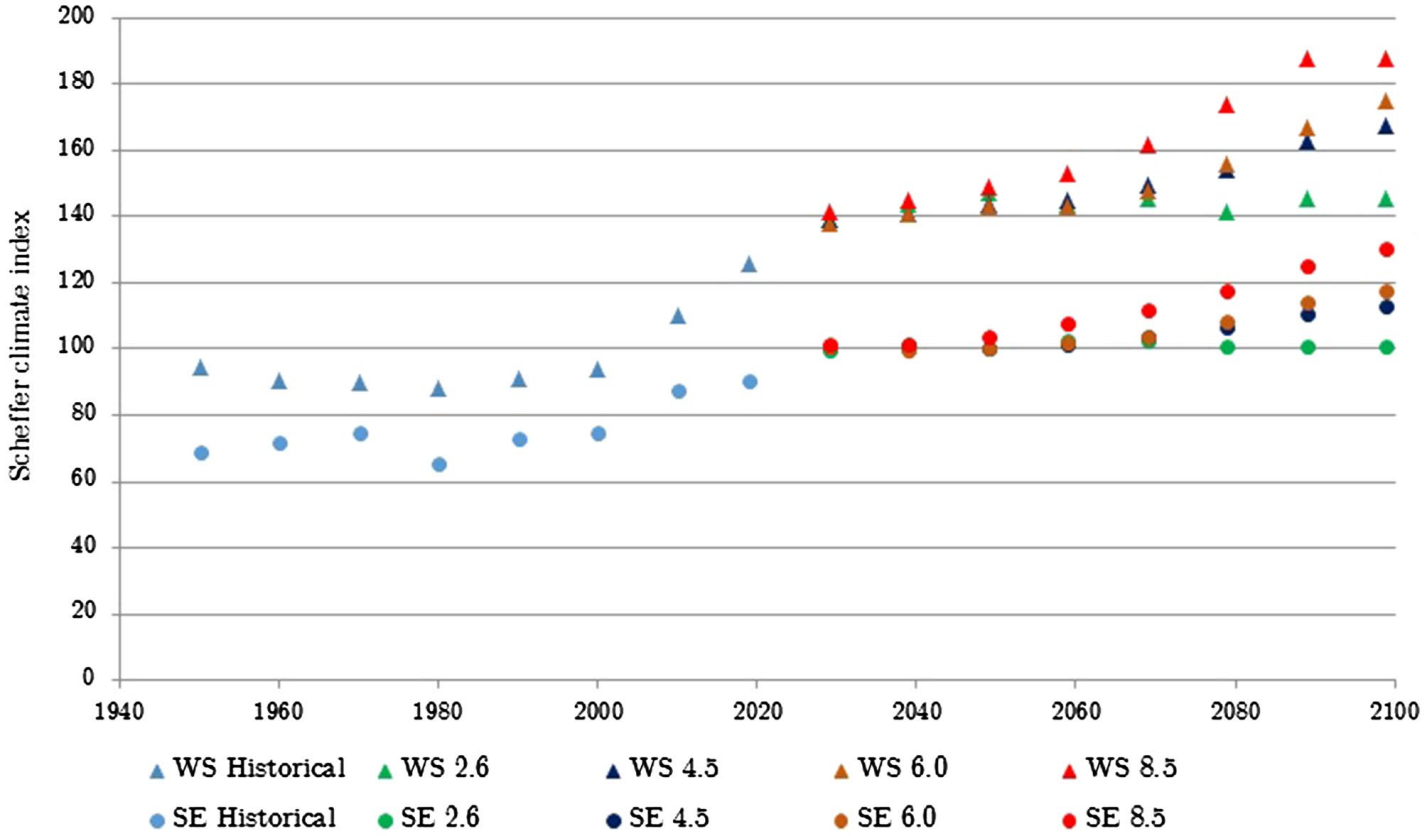
Actual and projected decadal SCI values for Western Scotland (WS) and South East England (SE) under RCP, 2.6, 4.5, 6.0 and 8.5 scenarios.

The future decadal SCI projections are shown in Fig. 5 for the West of Scotland and South East of England, representing regions with the highest and lowest SCI values, for RCP 2.6, 4.5, 6.0 and 8.5 scenarios. The graphical projections illustrate that under all scenarios there is a continuing projected increase in SCI over the next 10 to 20 years followed by stabilisation. Under the RCP 2.6 scenario this stabilisation continues until at least 2100 with no significant reduction. Under the other scenarios, however the stabilisation converts back in to a steady increase in index value after 2050 that continues until the end of the projection period (2100). Similar trends are seen for the other regions (shown graphically in the supplementary Figs S1–S8).

Traditionally SCI data is given as the tri-decadal value, and these values are shown in Table 2 for the noted time periods for all regions.

**Table 2:** Projected Tri-decadal (2100 value is bi-decadal) SCI values for all areas of the United Kingdom under RCP 2.6, 4.5, 6.0 and 8.5 scenarios.

Region	Tri-decadal Climate index						
	Current	RCP 2.6			RCP 4.5		
	2019	2050	2080	2100^a^	2050	2080	2100^a^
North Scotland	99	121	123	120	122	130	137
East Scotland	86	108	107	106	106	112	117
West Scotland	110	143	144	146	141	149	165
Northern Ireland	95	112	112	115	110	114	126
North West	96	111	114	112	111	117	127
North East	87	109	110	111	108	112	123
Midlands	86	103	104	105	99	103	113
East Anglia	87	104	105	105	102	106	114
South East	84	100	102	101	101	104	112
South West	105	121	123	121	117	121	133

	Current	RCP 6.0			RCP 8.5		
	2019	2050	2080	2100^a^	2050	2080	2100^a^
North Scotland	99	121	129	143	126	143	164
East Scotland	86	106	111	121	109	122	136
West Scotland	110	141	149	171	145	163	194
Northern Ireland	95	109	114	129	112	121	141
North West	96	111	117	131	114	125	143
North East	87	107	111	127	110	121	143
Midlands	86	99	103	116	101	110	128
East Anglia	87	101	106	117	103	112	127
South East	84	100	105	116	102	112	128
South West	105	116	121	137	119	129	150

^a^2100 value is bi-decadal.

The RCP 4.5 and 6.0 projections appear to be quite similar and may represent the most likely projections. RCP 8.5 was designed as a worst case scenario (often called the “business as usual scenario” but is now thought to be less probable due to measures already taken. RCP 2.6 relies on very stringent mitigation measures being taken so may also be less probable. The projections do indicate that even under the “least effect scenario” of RCP 2.6 all areas of the UK will move into the very high hazard category based on SCI.

The change in SCI values as shown in Table 2 demonstrate very well, the clear effect of the changing climate on the climate based decay hazard. Even with the lowest scenario which includes stringent mitigation efforts (RCP 2.6) an increase in decay hazard of between 16% to − 30% can be projected for all regions of the UK by the end of the century. With the RCP 6.0 emissions scenario the increase is in the range of 30% to 55% and 43% to 76% with the RCP 8.5 scenario.

It is interesting to note that although the climate projections suggest wetter and warmer winters and dryer and warmer summers, there is an increase in the SCI. This suggests that the warmer temperatures of the winter have more of an impact on total decay hazard than the dryer summers, which corresponds with the conclusions of Grontoft^16^.

### Implications of increasing hazard

As stated previously there are now doubts over the likelihood of the RCP 8.5 scenario being probable. However, the clear changing climate risk for decay may have a number of implications, the basic one of course being faster rates of decay. This could lead to economic loss due to higher maintenance costs and more frequent replacement of affected wood either for utility, safety or aesthetic reasons. There may also be cultural loss due to decay of historic wood structures and items.

However, it needs to be remembered that local effects will play major roles on whether or not a particular wooden item will decay. Situational variables such as local microclimates, prevailing winds and speed of drying are not taken into account with the broader climate index. Also the wood type and preservation or modification techniques used can significantly reduce decay risk.

The increasing climate based hazard should refocus scrutiny on building design and choice of durable woods. There may also be the increased need for effective and appropriate wood protection strategies via wood treatment’s and modification.

## Conclusions

There has been a clear increase in the climate index for decay in the United Kingdom from 1990 to the present day. This implies an associated rise in decay hazard. Current climate projections suggest that the UK will experience warmer and wetter winters and warmer and dryer summers. Calculations of the Scheffer Climate Index based on UK climate projections show that by 2050 a 10% to 30% increase in SCI is projected and by 2100 a possible 15% to 55% (dependent on region) increase could be observed.

The major implication is that to ensure serviceability of wood and wooden structures exposed to the environment, the use of good design, durable woods and properly treated or modified woods will be paramount.

## Methods

### Scheffer Climate Index

The Scheffer Climate Index SCI calculation is based on mean temperature and moisture content and uses the following equation

1 $$SCI=\frac{{\sum }_{Jan}^{Dec}\left[\left(T-2\right)\left(D-3\right)\right]}{16.7}$$

where, SCI = Scheffer Climate Index, T = mean monthly temperature, D = mean number of days in the month with 0.25 mm or more of rain. ${\sum }_{Jan}^{Dec}$ = the sum of the products for each month of the year.

The temperature and rain days have attached constants to accommodate minimum temperature requirements and to estimate wetting times. The divisor (16.7) is an arbitrary number designed to accommodate the index value approximately in a value range of 0–100, although values of 100 + are easily possible.

A general hazard risk has been assigned depending on the SCI values as follows; a value less than 35 indicating low hazard, values 35 to 65 as moderate hazard, 65 to 100 high hazard and above 100 as very high hazard.

### Historic and current Scheffer Climate Index determination

**Figure 6:**
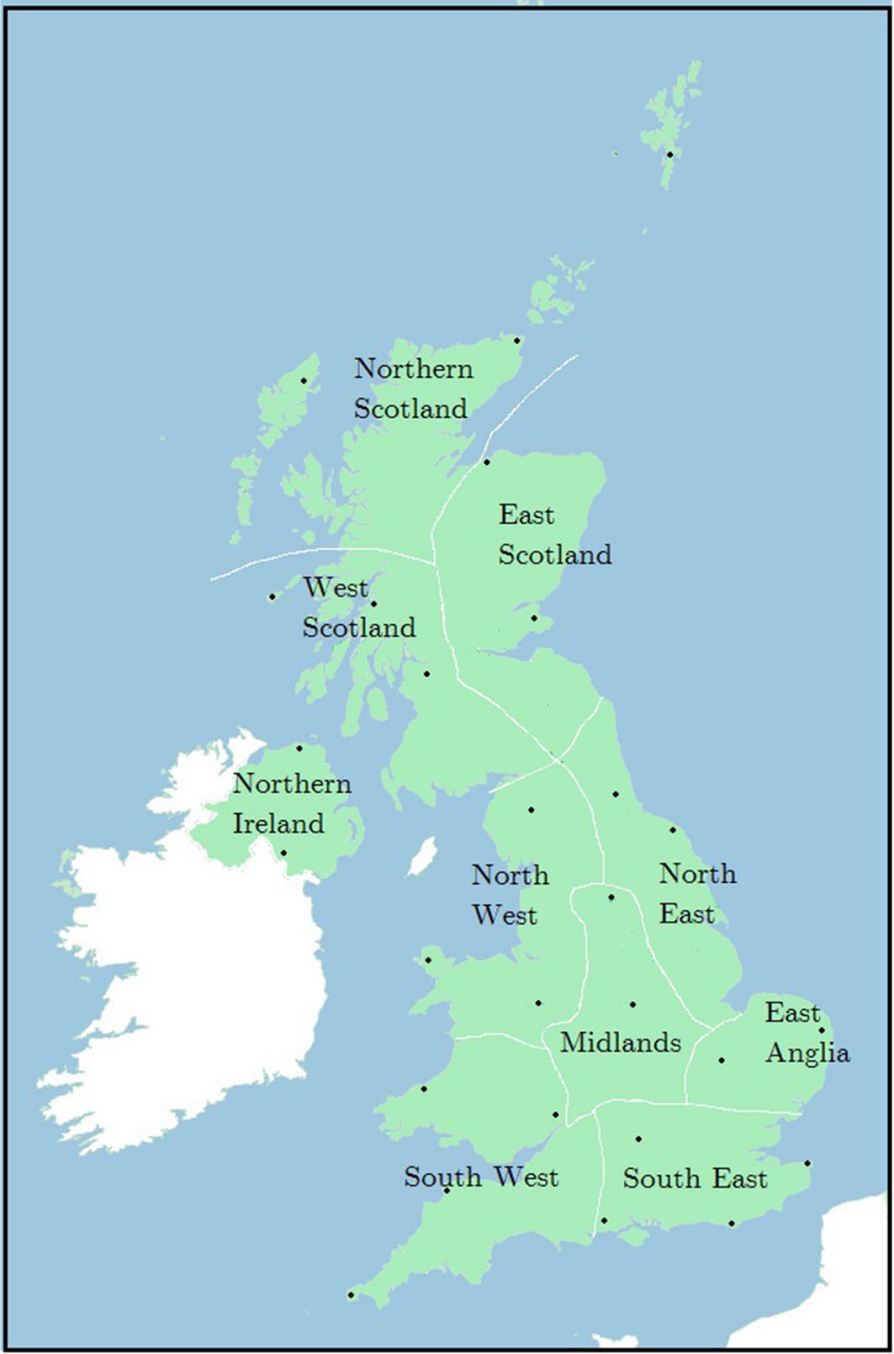
United Kingdom Regions (map created using Microsoft paint version 6.1).

Determination of the historic and current SCI was calculated using data from the UK Metrological office. For temperature, data was obtained from a number of weather stations around the UK that have publically available data, some of which goes back to the 1800’s. The station locations and associated regions are shown in Fig. 6 with identity and location data shown in supplementary material (Table S1). Monthly data was obtained from 1941 from at least 2 stations in each region, with further stations used where possible for more complete data. The data was obtained from the UK historic station data^27^. The data consists of values for the monthly mean maximum temperatures and mean minimum temperatures which were used to calculate the mean monthly temperature as required for the T-variable in Eq. (1).

The UK Metrological Office Hadley Centre observational data set (HadUKP data set)^28,29^, provides daily rainfall data over the same time periods on a regional level. The locations of stations were matched with the appropriate regions, and the number of days of rain greater than 0.25 mm determined for each month in the time period, providing the D-variable for Eq. (1).

The annual SCI was then calculated, for each station, using Eq. (1), with means calculated for each region. Mean SCI was then determined for decadal ranges and for tri-decadal– data from the 1960’s 1970s’ and 1980 were used to determine the mean 30 year SCI for 1990 and data from the 1990’s 2000’s and 2010’s (up to 2019) were used to determine the mean 30 (29) year SCI for 2019. To determine statistical relevance an F–test for variance followed by t-test for means (either assuming equal or unequal variance based on the F value) was performed on the mean data for pooled sites for each region (see supplemental data Table S2).

### Scheffer Climate Index projections

Projections of the future SCI value were made using the UK met office UK climate projections (UKCP 18) data. These data project the change in rainfall (in percentage terms) and change in mean temperature (in Celsius) on a regional administrative basis (see Fig. 6) for specific time periods at varying probability levels. The projections selected were those derived at the 50% probability level based on the RCP 2.6, RCP 4.5, RCP 6.0 and RCP 8.5 scenarios.

Using the current (2009 to 2019) mean temperatures and rain fall, as calculated from the historical data sets previously described as the base point, the required climate variables, for each station, area and time period, were calculated as follows. The projected change in temperature was added to the current temperature data to obtain projected temperatures. Similarly, the projected percentage change in rain fall was applied to the mean distribution of the current rainfall data for each location and month to obtain projected rainfall values, assuming a similar future distribution. The projected SCI was then calculated, using Eq. (1), at decadal and tri-decadal intervals under all RCP scenarios. Mean values of each area were calculated using the data from the stations within each area.

## Supplementary information

Supplementary Information.

## Data Availability

Original climate datasets used are available from the UK metrological office. The datasets generated during and/or analysed during the current study are available from the corresponding author on reasonable request.
