# Supplementary material for: Observed and projected changes in the climate based decay hazard of timber in the United Kingdom
Source: Sci Rep. 2020 Oct 1;10:16287. doi: 10.1038/s41598-020-73239-1 (PMC7531001; doi:10.1038/s41598-020-73239-1)
Supplement: Supplementary file 1 — Supplementary Information. [file 41598_2020_73239_MOESM1_ESM.pdf]

Observed and projected changes in the climate based decay hazard of timber in the United Kingdom

Curling S.F*, Ormondroyd G.A.'

BioComposites Centre, Bangor University, Bangor, Gwynedd, LL57 2UW, United Kingdom
s.curling@bangor.ac.uk, g.ormondroyd@bangor.ac.uk

Supplementary Information

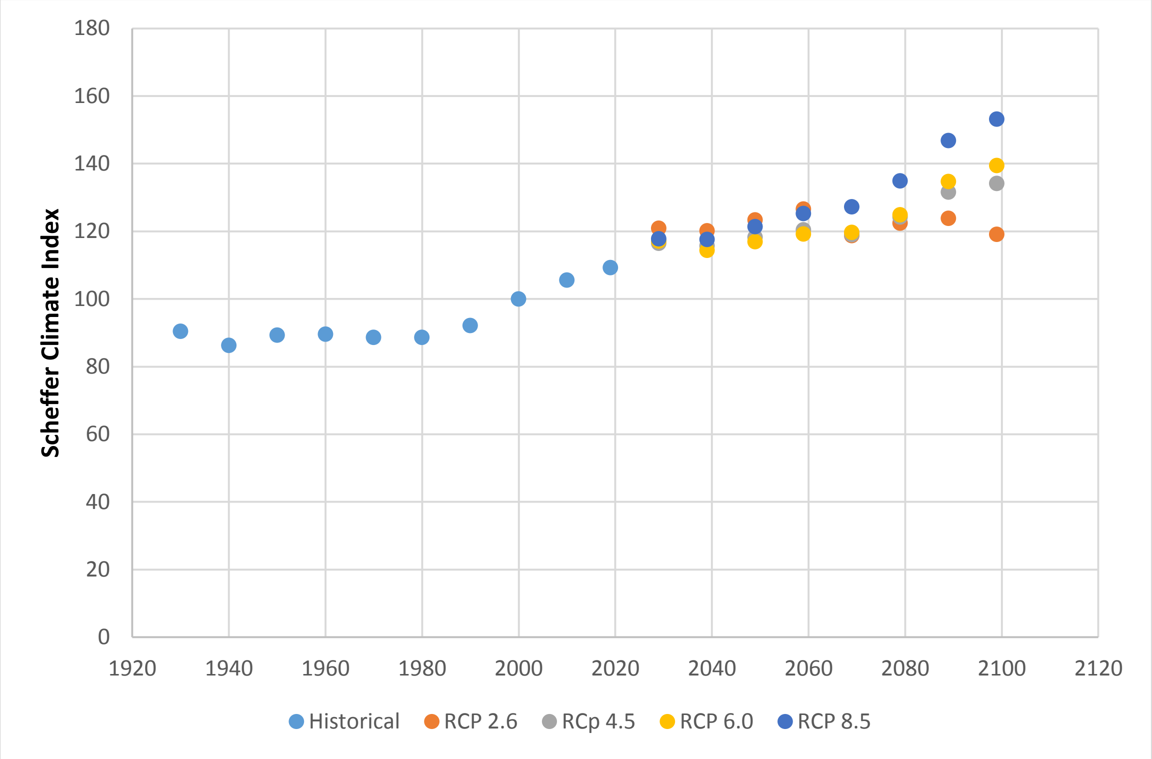

Figure S1 Actual and projected decadal SCI values for the South West region under RCP, 2.6, 4.5, 6.0 and 8.5 scenarios

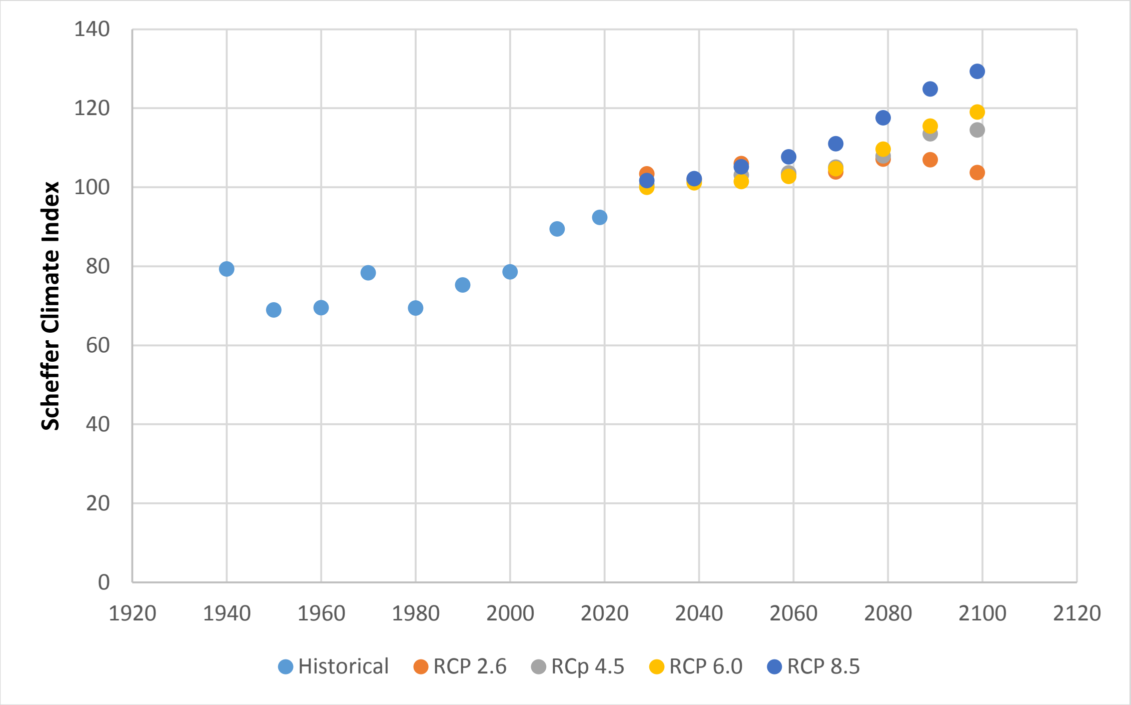

Figure S2 Actual and projected decadal SCI values for the East Anglia region under RCP, 2.6, 4.5, 6.0 and 8.5 scenarios

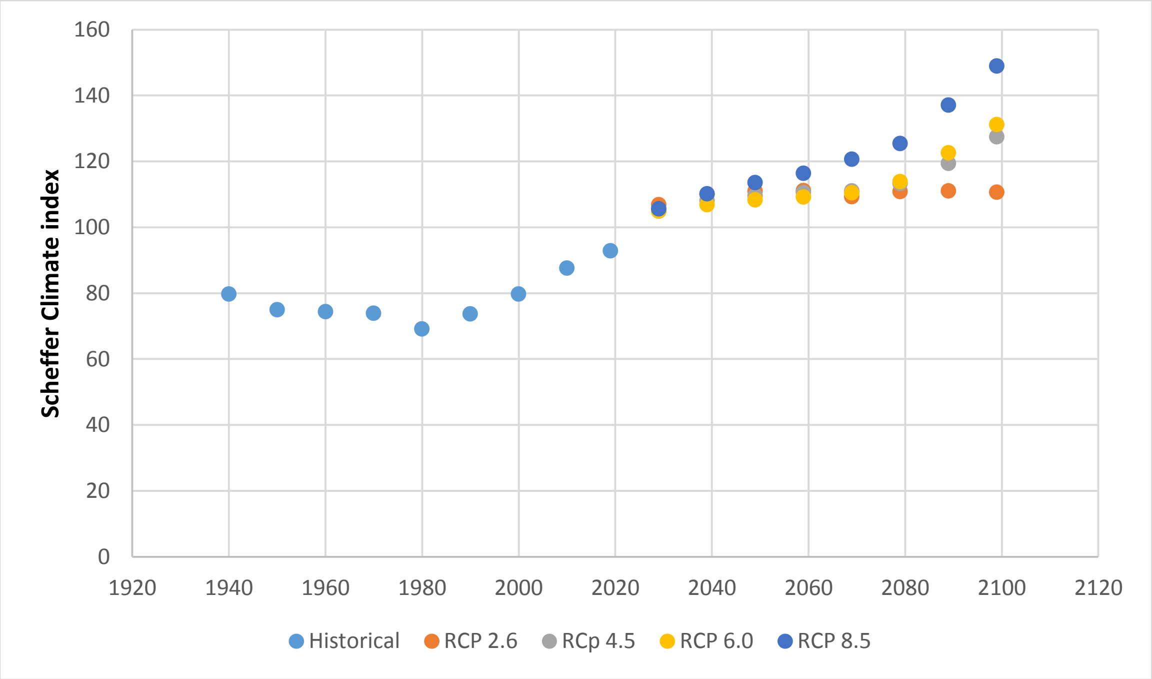

Figure S3 Actual and projected decadal SCI values for the North East region under RCP, 2.6, 4.5, 6.0 and 8.5 scenarios

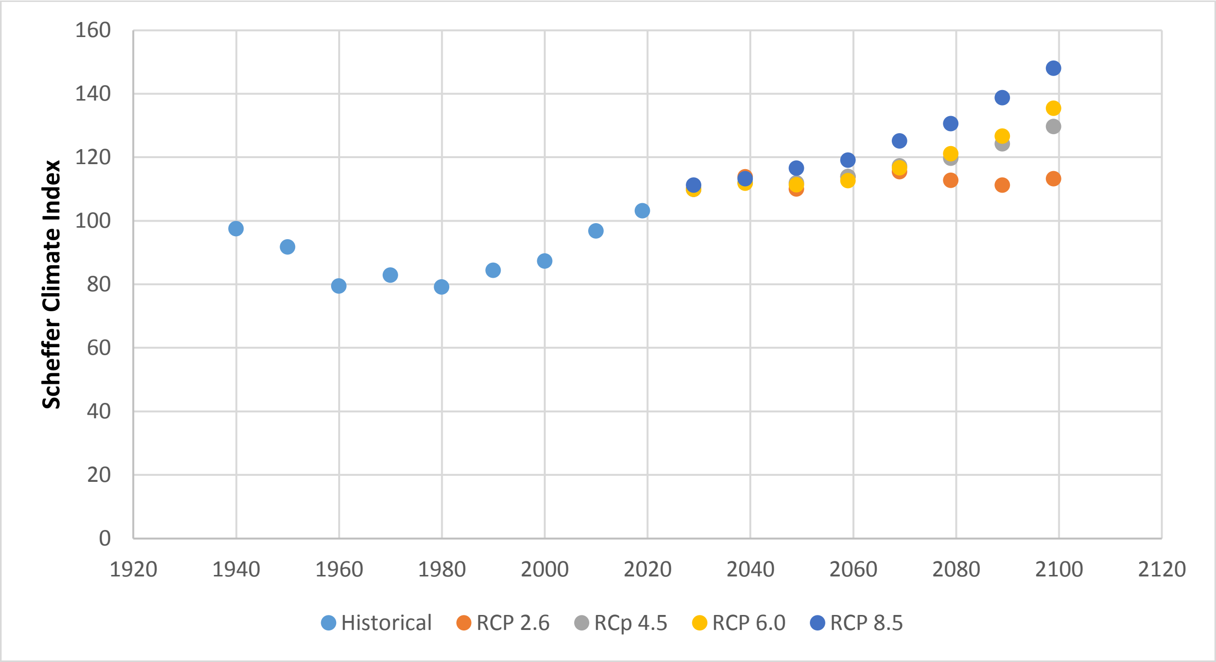

Figure S4 Actual and projected decadal SCI values for the North West region under RCP, 2.6, 4.5, 6.0 and 8.5 scenarios

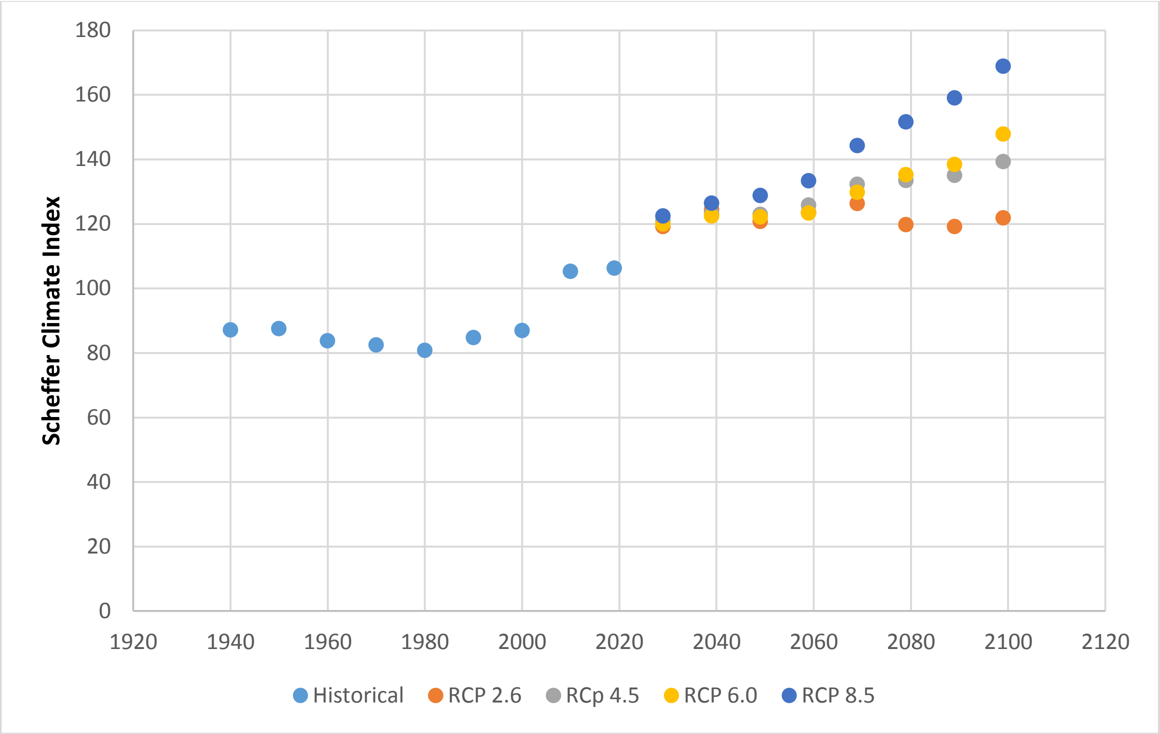

Figure S5 Actual and projected decadal SCI values for the North Scotland region under RCP, 2.6, 4.5, 6.0 and 8.5 scenarios

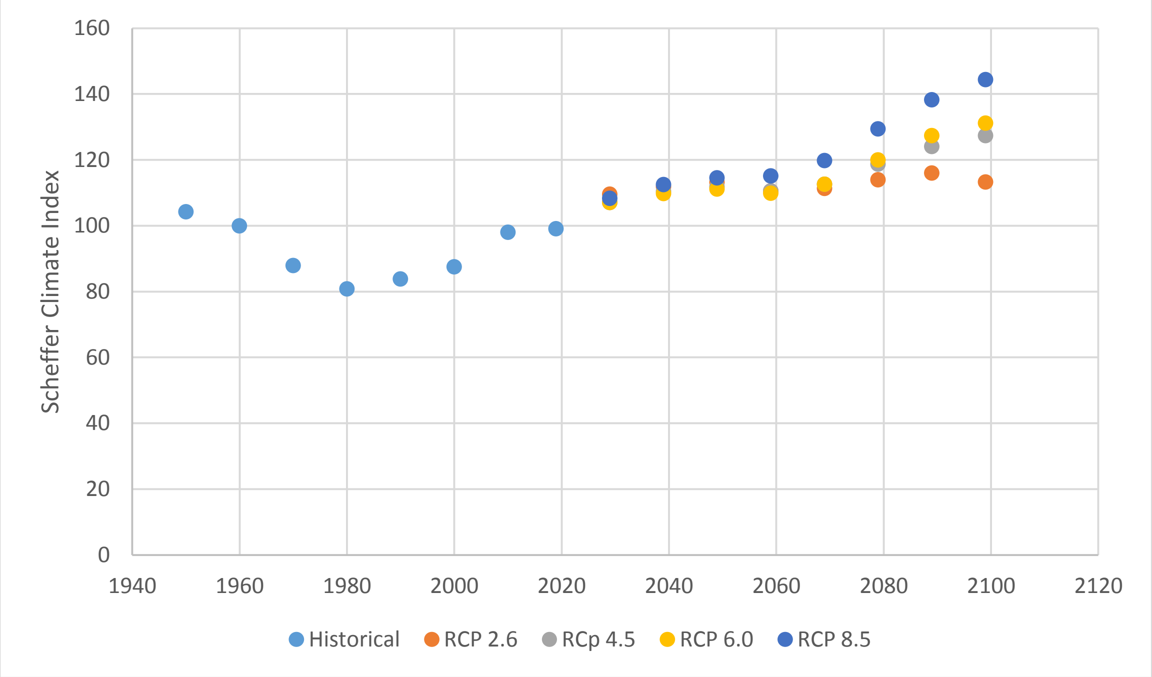

Figure S6 Actual and projected decadal SCI values for the Northern Ireland region under RCP, 2.6, 4.5, 6.0 and 8.5 scenarios

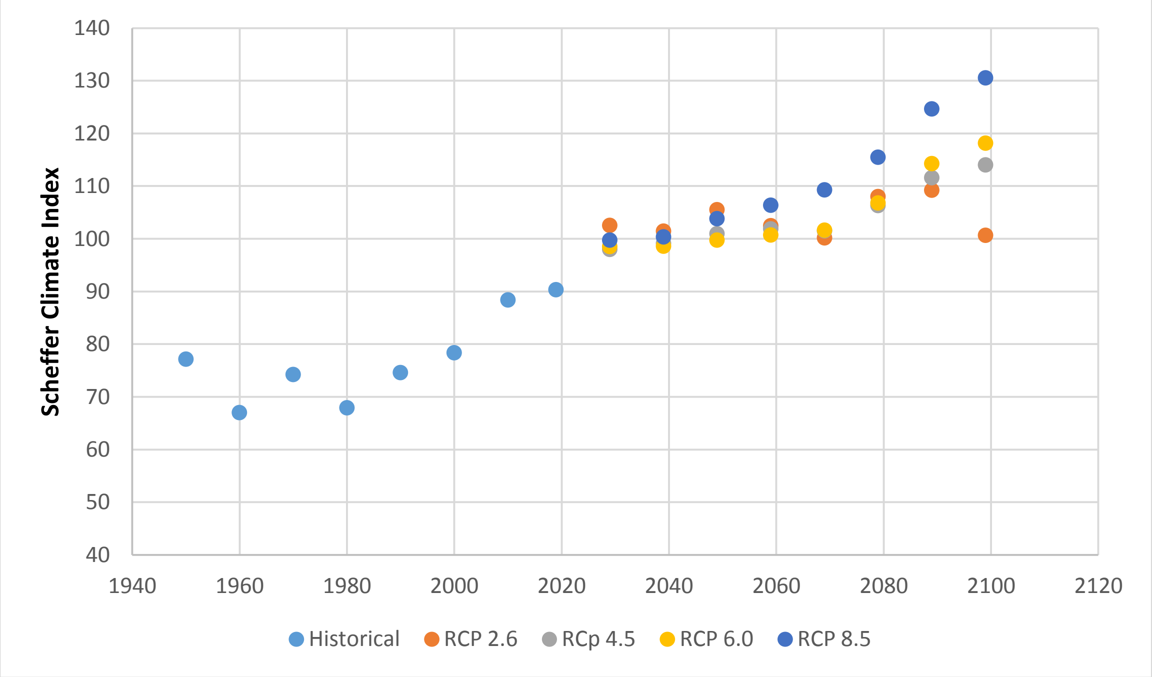

Figure S7 Actual and projected decadal SCI values for the Midlands region under RCP, 2.6, 4.5, 6.0 and 8.5 scenarios

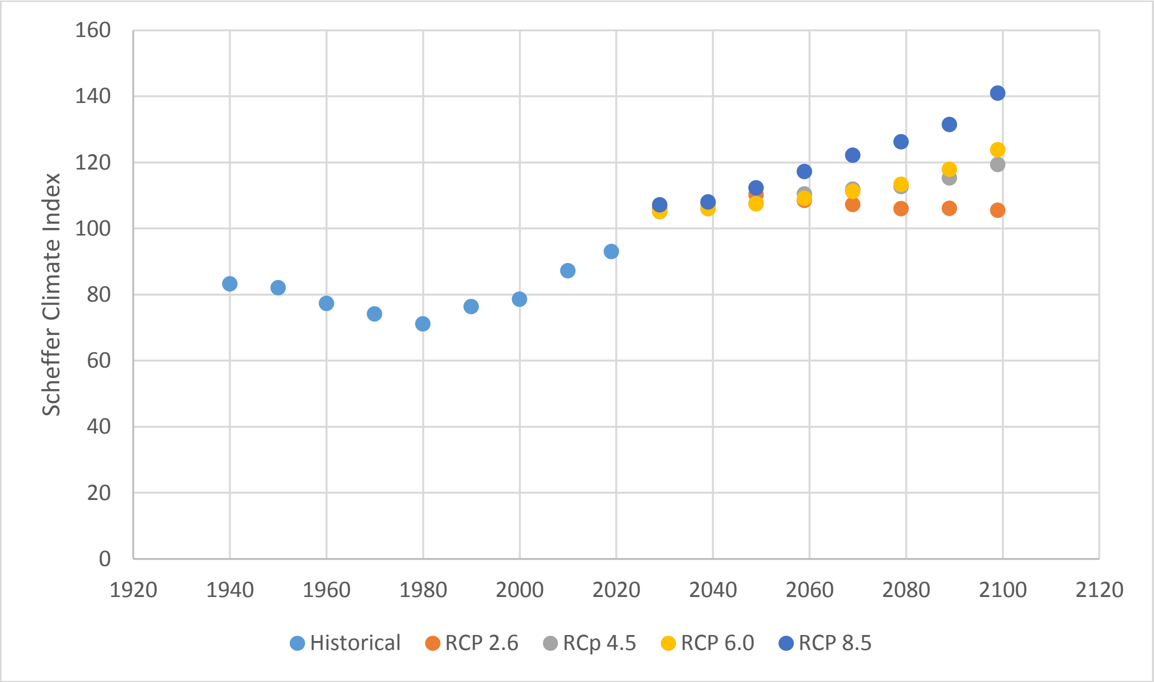

Figure S8 Actual and projected decadal SCI values for the East Scotland region under RCP, 2.6, 4.5, 6.0 and 8.5 scenarios

Table S1. Weather station identification and location data

Station	Region	Longitude	latitude	Height above sea level (m)
Stornaway	North Scotland	-6.318	58.214	15
Wick		-3.088	58.54	36
Lerwick		-1.183	60.139	82
Leuchars	East Scotland	-2.861	56.377	10
Nairn		-3.821	57.593	23
Paisley	West Scotland	-4.43	55.846	32
Dunstaffnage		-5.439	56.451	3
Tiree		-6.880	56.5	12
Armagh	Northern Ireland	-6.649	54.352	62
Ballypatrick Forest		-6.153	55.181	156
Newton Rigg	North West England and North Wales	-2.786	54.67	169
Shawbury		-2.663	52.794	72
Valley		-4.535	53.252	10
Durham	North East England	-1.585	54.768	102
Whitby		-0.624	54.481	60
Sutton Bonnington	Midlands	-1.250	52.833	48
Bradford		-1.722	53.813	134
Cambridge	East Anglia	0.102	52.245	26
Lowestoft		1.727	52.483	18
Eastbourne	South East and South Central England (SE)	0.285	50.762	7
Hurn		-1.835	50.779	10
Manston		1.337	51.346	49
Oxford		-1.262	51.761	63
Aberporth	South Wales and South West England (SW)	-4.57	52.139	133
Camborne		-5.327	50.218	87
Ross-on-Wye		-2.584	51.911	67

Table S2 Statistical summary of differences in regional tridecadal Scheffer Climate indices between 1990 and 2019.

Region	F crit	F value	Equality of variance	T-test p value
North Scotland	3.44	2.80	Equal	0.0027
East Scotland	5.05	5.19	Unequal	0.0046
West Scotland	3.44	3.02	Equal	0.0326
Northern Ireland	5.05	1.20	Equal	0.0841
North West	3.44	1.60	Equal	0.0193
North East	5.05	6.18	Unequal	0.0016
Midlands	5.05	2.37	Equal	0.0015
East Anglia	5.05	2.68	Equal	0.0033
South East	2.81	1.77	Equal	0.0005
South West	3.44	1.89	Equal	0.0008
